# Supplementary material for: Acceptability of the COVID-19 Vaccine and Its Determinants among University Students in Saudi Arabia: A Cross-Sectional Study
Source: Vaccines (Basel). 2021 Aug 25;9(9):943. doi: 10.3390/vaccines9090943 (PMC8473185; doi:10.3390/vaccines9090943)
Supplement: Supplementary file 1 [file vaccines-09-00943-s001.zip › vaccines-1322111-supplementary.pdf]

Supplementary Material

Supplementary file1: Questionnaire of acceptability of the COVID-19 vaccine

I agree to participate in this study

1 ☐ No (you may stop here, thank you)

2 ☐ Yes (please start the questionnaire)

PART ONE: Background Information

1. Your gender?

1 ☐ Male

2 ☐ Female

2. Your age? (________________________)

3. Your marital status?

1 ☐ Not-married

2 ☐ Married

4. Discipline of study?

1 ☐ Non-health related

2 ☐ Health-related

5. Education level?

1 ☐ Year 1

2 ☐ Middle years

3 ☐ Final year

6. Have you contracted COVID-19?

1 ☐ No

2 ☐ Yes

7. Have any of your family members or friends contracted COVID-19?

1 ☐ No

2 ☐ Yes

8. Have any of your family members or friends died due to COVID-19?

1 ☐ No

2 ☐ Yes

9. Do you regularly receive the flu vaccine?

1 ☐ No

2 ☐ Yes

10. Have you booked an appointment to receive the COVID-19 vaccine?

1 ☐ No

2 ☐ Yes

3 ☐ I received the COVID-19 vaccine Skip to question 14

PART TWO: Acceptability of the COVID-19 vaccines

11. I trust the COVID-19 vaccines that provided in Saudi Arabia.

1 ☐ Strongly Agree
2 ☐ Agree
2 ☐ Neutral
2 ☐ Disagree
2 ☐ Strongly Disagree

12. Factors influencing trust toward the COVID-19 vaccines provided in Saudi Arabia:

	Strongly Agree	Agree	Neutral	Disagree	Strongly Disagree
**Confidence in the government**	☐	☐	☐	☐	☐
**Confidence in the healthcare system**	☐	☐	☐	☐	☐
**Experience and reputation of manufacturers**	☐	☐	☐	☐	☐
**Scientific information about vaccines**	☐	☐	☐	☐	☐
**World Health Organization public advice on vaccines**	☐	☐	☐	☐	☐
**Important community figures who received the vaccine**	☐	☐	☐	☐	☐

13. I will receive the COVID-19 vaccine when given the opportunity.

1 ☐ No Skip to question 15
2 ☐ Yes

14. Motives to receive the COVID-19 vaccine:

	Strongly Agree	Agree	Neutral	Disagree	Strongly Disagree
**Prevention purposes**	☐	☐	☐	☐	☐
**Influence from my family**	☐	☐	☐	☐	☐
**Influence from friends**	☐	☐	☐	☐	☐
**Influence from media**	☐	☐	☐	☐	☐
**Public awareness information on the vaccine**	☐	☐	☐	☐	☐
**Influence from community figures**	☐	☐	☐	☐	☐
**Confidence in the vaccines' science and safety**	☐	☐	☐	☐	☐

15. Factors that drive the refusal of COVID-19 vaccine:

	Strongly Agree	Agree	Neutral	Disagree	Strongly Disagree
**Expedited vaccine trials due to short time**	☐	☐	☐	☐	☐
**Concerns regarding long-term side effects**	☐	☐	☐	☐	☐
**The COVID-19 vaccine is an unnecessary procedure**	☐	☐	☐	☐	☐
**Preventive measures are enough for combating COVID-19**	☐	☐	☐	☐	☐
**Influence from anti-vaccine movement**	☐	☐	☐	☐	☐
**General lack of trust in the COVID-19 vaccine**	☐	☐	☐	☐	☐

In the space below, please write any additional notes or information that you believe will enhance the results of the study.

THANK YOU FOR COMPLETING THIS SURVEY.

أستبانة حول قابلية لقاح كوفيد-19

أوافق على المشاركة في هذه الدراسة

1 ☐ لا (لطفًا بإمكانك التوقف هنا، شكرًا لك)

2 ☐ نعم (من فضلك ابدأ الإجابة على اسئلة الاستبانة)

الجزء الأول: معلومات أساسية

1. جنسك؟

1 ☐ ذكر

2 ☐ أنثى

2. عمرك؟ (____________________)

3. الحالة الاجتماعية؟

1 ☐ غير متزوج

2 ☐ متزوج

4. التخصص الدراسي؟

1 ☐ تخصص غير صحي

2 ☐ تخصص صحي

5. المستوى الدراسي؟

1 ☐ السنة الدراسية الأولى

2 ☐ ما بين السنة الأولى والسنة الأخيرة

3 ☐ السنة الدراسية الأخيرة

6. هل سبق إصابتك بكوفيد-19؟

1 ☐ لا

2 ☐ نعم

7. هل سبق إصابة أحد أقاربك أو أصدقائك بكوفيد-19؟

1 ☐ لا

2 ☐ نعم

8. هل توفي أحد أقاربك أو أصدقائك جراء الإصابة بكوفيد-19؟

1 ☐ لا

2 ☐ نعم

9. هل تحرص على تلقي لقاح الأنفلونزا الموسمية بانتظام؟

1 ☐ لا

2 ☐ نعم

10. هل حجزت موعد لتلقي لقاح كوفيد-19؟

1 ☐ لا

2 ☐ نعم

3 ☐ سبق لي تلقي لقاح كوفيد-19 اذهب للسؤال 14

الجزء الثاني: قابلية لقاحات كوفيد-19

11. أثق في لقاحات كوفيد-19 المستخدمة في المملكة العربية السعودية.

1 ☐ أتفق بشدة

2 ☐ أتفق

3 ☐ محايد

4 ☐ لا أتفق

5 ☐ لا أتفق بشدة

12. عوامل ثقتي في لقاحات كوفيد-19 المستخدمة في المملكة العربية السعودية:

	أتفق بشدة	أتفق	محايد	لا أتفق	لا أتفق بشدة
**1. الثقة في الحكومة**	☐	☐	☐	☐	☐
**2. الثقة في نظام الرعاية الصحية**	☐	☐	☐	☐	☐
**3. خبرة وسمعة الجهات المصنعة للقاحات**	☐	☐	☐	☐	☐
**4. المعلومات العلمية المتوفرة عن اللقاحات**	☐	☐	☐	☐	☐
**5. النصائح العامة من منظمة الصحة العالمية بشأن اللقاحات**	☐	☐	☐	☐	☐
**6. الشخصيات المجتمعية الهامة التي تلقت اللقاح**	☐	☐	☐	☐	☐

13. سأتلقى لقاح كوفيد-19 عندما تتاح لي الفرصة.

1 ☐ لا انتقل للسؤال 15

2 ☐ نعم

14. الدوافع لتلقي لقاح كوفيد-19.

	أتفق بشدة	أتفق	محايد	لا أتفق	لا أتفق بشدة
**1. الرغبة في الوقاية من الوباء**	☐	☐	☐	☐	☐
**2. تأثير من عائلتي**	☐	☐	☐	☐	☐
**3. تأثير الاصدقاء**	☐	☐	☐	☐	☐
**4. تأثير الإعلام**	☐	☐	☐	☐	☐
**5. المعلومات التوعوية المتوفرة حول اللقاح**	☐	☐	☐	☐	☐
**6. تأثير من الشخصيات المجتمعية**	☐	☐	☐	☐	☐
**7. الثقة في علم وسلامة اللقاحات**	☐	☐	☐	☐	☐

14. العوامل المانعة من تلقي لقاح كوفيد-19.

	أتفق بشدة	أتفق	محايد	لا أتفق	لا أتفق بشدة
**1. تجارب اللقاح المعجلة بسبب ضيق الوقت**	☐	☐	☐	☐	☐
**2. الخشية من الآثار الجانبية للقاح على المدى البعيد**	☐	☐	☐	☐	☐
**3. لقاح كوفيد-19 يعتبر إجراء غير ضروري**	☐	☐	☐	☐	☐
**4. التدابير الوقائية كافية لمكافحة كوفيد-19**	☐	☐	☐	☐	☐
**5. تأثير الحملات المضادة للتطعيم**	☐	☐	☐	☐	☐
**6. انعدام الثقة بشكل عام في تطعيمات كوفيد-19**	☐	☐	☐	☐	☐

في المساحة أدناه، فضلاً أكتب أي ملاحظات أو معلومات إضافية تعتقد أنها ستعزز من نتائج الدراسة.

شكرًا لك للمشاركة في هذه الاستبانة
